# Biomimetic peptide self-assembly: interfacing with biomacromolecules to regulate cellular signaling

**DOI:** 10.1038/s12276-026-01691-6

**Published:** 2026-04-14

**Authors:** Dohyun Kim, Gaeun Park, Min-Seok Seu, Injun Hwang, Sanjay Kumar Perumandla, Jaemo Lee, Ja-Hyoung Ryu

**Affiliations:** 1 Department of Chemistry, Ulsan National Institute of Science and Technology (UNIST), Ulsan, Republic of Korea; 2 GIST InnoCORE AI-Nano Convergence Institute for Early Detection of Neurodegenerative Diseases, Gwanjgu Institute of Science and Technology, Gwangju, Republic of Korea

**Keywords:** Nanostructures, Bioinspired materials

## Abstract

Supramolecular self-assembly represents a spontaneous and reversible process that bridges discrete molecular building blocks with nanoscale architecture through non-covalent interactions. By rationally tuning these interactions, diverse nanostructures can be precisely constructed, each exhibiting distinct physicochemical and functional properties. The dynamic and multivalent nature of supramolecular assemblies endows them with structural adaptability and cooperative binding, enabling responsiveness to environmental cues and amplification of weak molecular interactions. Nature provides abundant paradigms for such self-organization, in which organized supramolecular interfaces mediate complex biological functions. Inspired by these natural principles, artificial self-assembly systems have been engineered to emulate the hierarchical organization and functional adaptability of living systems. In this Review, we summarize recent advances in nature-inspired supramolecular assemblies, focusing on peptide-based systems that exploit the chemical diversity of amino acids to modulate biomacromolecular interactions and cellular signaling. Understanding these biomimetic design principles offers a foundation for developing next-generation functional materials that bridge molecular precision with biological functionality.

## Peptide-based systems transform biomacromolecular interactions

This article explores how scientists are using the natural process of supramolecular self-assembly to create new materials that can interact with biological systems. Supramolecular self-assembly is a process in which molecules come together to form larger structures without forming strong chemical bonds. The study addresses how these assemblies can be used to design materials that mimic natural processes in cells. Researchers have focused on creating peptide-based assemblies. These assemblies can interact with cell membranes, proteins, and nucleic acids to influence cellular functions. The study uses various methods to design these assemblies, such as modifying peptide sequences to target specific cell components such as mitochondria or lysosomes. The results show that these peptide assemblies can disrupt cell membranes or interfere with protein interactions, leading to effects such as cell death in cancer cells.

This summary was initially drafted using artificial intelligence, then revised and fact-checked by the author.

## Introduction

Supramolecular self-assembly represents a spontaneous and reversible equilibrium process bridging discrete molecular building blocks and nanoscale architectures. Through rational building blocks design, the balance of non-covalent interactions such as electrostatic forces, dipole–dipole interactions, van der Waals forces, hydrogen bonding, and π–π stacking can be finely tuned^1^. Such subtle modulation of intermolecular interactions allows precise control over the underlying free-energy landscape and the assembly pathway. Depending on these intermolecular forces and environmental conditions, supramolecular assemblies can form various nanostructures. Specifically, the pathway complexity often leads to multiple assembly products, including thermodynamically stable, kinetically trapped, or metastable nanostructures. This pathway selection is particularly important in bottom-up fabrication, enabling the construction of hierarchically organized nanostructures, including nanofibers, nanospheres, nanotubes, and nanovesicles^2–5^. Importantly, these supramolecular nanostructures, which differ in morphology and stability, are often associated with distinct physicochemical properties and biological or material functions. Therefore, a comprehensive understanding of self-assembly principles is essential for the rational design of next-generation functional materials, bridging molecular-level interactions with emergent macroscopic properties.

Supramolecular assemblies exhibit unique functional properties that cannot be achieved by individual molecular components. Their inherent dynamic property enables structural adaptability and functional responsiveness, whereas the cooperative clustering of functional epitopes within assemblies gives rise to multivalent interactions that substantially enhance binding avidity and specificity^6–10^. The dynamic nature of supramolecular assemblies has been well demonstrated through Förster resonance energy transfer (FRET) experiments^11^. In a representative study, two self-assembled molecular building blocks were separately labeled with an FRET donor (Cy3) and an acceptor (Cy5). Upon mixing, a pronounced increase in FRET efficiency was observed over time, revealing that monomeric constituents can dynamically exchange between assemblies. This dynamic monomer exchange highlights the intrinsic reversibility and dynamic property of supramolecular systems, which are essential for adaptive functions in complex biological environments. Beyond their dynamics, the multivalent nature of supramolecular assemblies has a crucial role in amplifying molecular recognition. The individual monomers typically exhibit low affinity toward their substrates, whereas their assembled forms achieve markedly enhanced binding through multivalent interactions. Similarly, nanoparticle-based supramolecular scaffolds have been shown to facilitate multivalent recognition of cellular receptors, demonstrating how spatial organization at the nanoscale can strengthen weak molecular interactions^12–15^.

At the mechanistic level, multivalency arises from the spatial organization of multiple binding motifs within a supramolecular assembly, which fundamentally alters the thermodynamics and kinetics of molecular recognition^16–18^. In monomeric systems, individual peptide–target interactions are typically transient and characterized by low affinity owing to limited contact area and rapid dissociation. By contrast, supramolecular assemblies present multiple binding epitopes in a confined and preorganized manner, effectively increasing the local concentration of ligands and enabling simultaneous or sequential binding events. This spatial confinement reduces the entropic penalty associated with rebinding and markedly decreases the effective dissociation rate, resulting in enhanced binding avidity rather than a simple sum of individual affinities. Moreover, cooperative interactions emerge when initial binding events promote subsequent interactions by stabilizing the assembly–target interface or inducing local conformational alignment. Such cooperativity enables weak individual interactions to be amplified into stable, high-avidity associations that are highly sensitive to target density and spatial distribution. Consequently, assembled peptide architectures preferentially engage targets that are densely clustered. This density-dependent recognition enables selective binding to high-density targets, a capability that is largely inaccessible to monomeric peptides or small molecules that interact in a one-to-one manner. This mechanistic distinction underlies the superior performance of supramolecular assemblies in engaging lipid membranes, proteins, and nucleic acids within complex cellular environments. Taken together, the unique supramolecular property underscores the fundamental advantages of self-assembly over discrete monomeric components.

In nature, self-assembled nanostructures are widely exploited to regulate cellular functions owing to their unique combination of dynamic adaptability and multivalent cooperativity across molecular and supramolecular scales^19,20^. A representative example is the folding and hierarchical assembly of proteins, which are composed of simple amino acid building blocks yet spontaneously adopt thermodynamically stable tertiary and quaternary structures. Although the linear primary sequence of amino acids has limited capacity to interact with biomacromolecules, the folded higher-order architectures expose organized functional epitopes that enable extensive intermolecular contacts, commonly referred to as protein–protein interactions (PPIs), through large self-assembled biointerfaces. These PPIs typically occur over substantial interfacial areas (~300–3,000 Å²) and are stabilized by a delicate balance of non-covalent forces, including electrostatic attraction, hydrogen bonding, and hydrophobic interactions^21,22^. Such supramolecular organized interfaces are crucial for mediating a wide spectrum of biological processes, ranging from enzymatic catalysis and signal transduction to transcriptional regulation and cytoskeletal organization. Moreover, the intrinsic dynamic and reversible nature of these supramolecular interactions allows PPIs to function as molecular switches, enabling precise spatial and temporal regulation of cellular signaling, differentiation, and metabolic pathways^23^. Indeed, this dynamic cooperativity represents one of nature’s most powerful design strategies, allowing biological assemblies to form, dissociate, and reorganize in response to environmental stimuli or molecular feedback. Understanding these natural principles provides valuable inspiration for designing biomimetic supramolecular assemblies that can emulate the adaptive and regulatory functions of living systems.

**Fig. 1:**
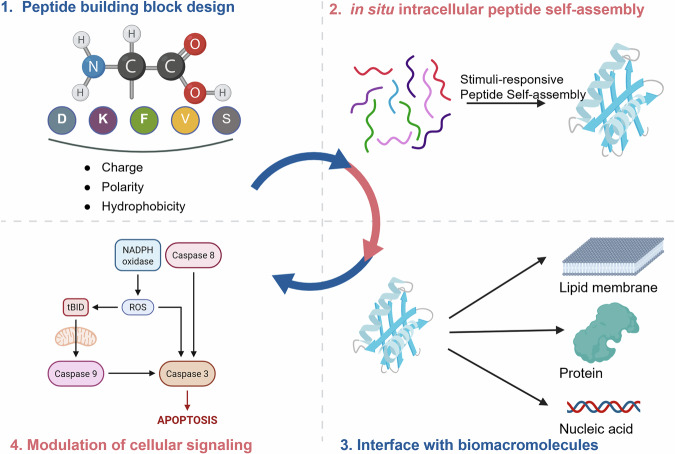
Schematic illustration summarizing this Review. Peptide building blocks were designed through rational molecular design principles. In response to cellular stimuli, intracellular peptide self-assembly can be achieved. The resulting biomimetic peptide assemblies interface with various biomacromolecules, including lipid membranes, proteins, and nucleic acids, thereby modulating cellular signaling pathways. NADPH nicotinamide adenine dinucleotide phosphate, ROS reactive oxygen species, tBID truncated BH3 interacting domain death agonist. The figure was created with BioRender.com.

In this Review, we summarize recent advances in nature-inspired artificial self-assembly systems that mimic the hierarchical organization and functional dynamics observed in living organisms. Guided by the principles of natural self-assembly, bioactive materials have been engineered over the past decades as powerful tools to regulate cellular signaling through interactions with various biomacromolecules, including proteins, lipid membranes, and nucleic acids. Specifically, various classes of self-assembling systems, including polymeric, lipid-based, and DNA-based materials, have been extensively explored as biomimetic platforms for regulating cellular processes. Polymeric assemblies offer high structural stability and tunable mechanical properties; however, their relatively limited molecular precision and batch-to-batch heterogeneity often restrict fine control over biomacromolecular interfaces^24,25^. Lipid-based assemblies, such as liposomes or lipid nanoparticles, closely resemble biological membranes and exhibit excellent biocompatibility, yet their functional diversity is largely constrained by lipid composition and often relies on additional functionalization to achieve signaling specificity^26,27^. DNA-based assemblies provide programmability and structural predictability through base-pairing interactions, but their susceptibility to nuclease degradation and limited chemical diversity can hinder long-term intracellular applications^28,29^. By contrast, peptide-based self-assembly has emerged as a particularly attractive platform owing to its intrinsic biocompatibility and its remarkable tunability derived from the combinatorial diversity of 20 amino acids^30–37^. Depending on their chemical properties, such as charge, polarity, and hydrophobicity, specific amino acid residues can be strategically incorporated to endow the resulting supramolecular structures with desired biological functions. Moreover, peptides inherently mimic protein interfaces, allowing assembled structures to recapitulate key features of natural biomolecular interactions, such as multivalency, adaptability, and dynamic reversibility. Considering these unique advantages, this Review focuses exclusively on peptide-based self-assembly, highlighting how such systems interact with biomacromolecules to modulate cellular signaling pathways in a nature-mimetic manner (Fig. 1).

## Biomimetic peptide self-assembly interfacing with lipid bilayer to regulate cellular signaling

The lipid bilayer is a fundamental biomacromolecule that serves as a biological barrier separating the intracellular and extracellular environments. It is primarily composed of amphiphilic phospholipids, cholesterol, glycolipids, and membrane proteins, which spontaneously self-assemble in aqueous environments. During this process, the hydrophobic acyl chains of lipids orient toward the interior to minimize contact with water, whereas the hydrophilic head groups face the aqueous surroundings, forming a stable bilayer structure. This self-assembly process is thermodynamically driven by the minimization of interfacial free energy and stabilized through van der Waals, electrostatic, and hydrogen-bonding interactions among lipid molecules^38,39^.

Lipid bilayers are ubiquitous throughout cellular architecture, forming the structural basis of both the plasma membrane and the membranes surrounding intracellular organelles such as the mitochondria, endoplasmic reticulum (ER), Golgi apparatus, and lysosomes. Despite sharing a common bilayer framework, each organelle exhibits distinct lipid compositions and membrane protein distributions that endow specialized physicochemical and biological properties^40,41^. For instance, mitochondrial membranes contain cardiolipin, essential for oxidative phosphorylation and energy transduction; ER membranes are enriched with unsaturated phospholipids that maintain membrane fluidity for protein synthesis and folding; Golgi membranes possess unique sphingolipids and glycosylated components critical for vesicular trafficking; and lysosomal membranes are specialized with glycoproteins and cholesterol to maintain stability in acidic environments.

**Fig. 2:**
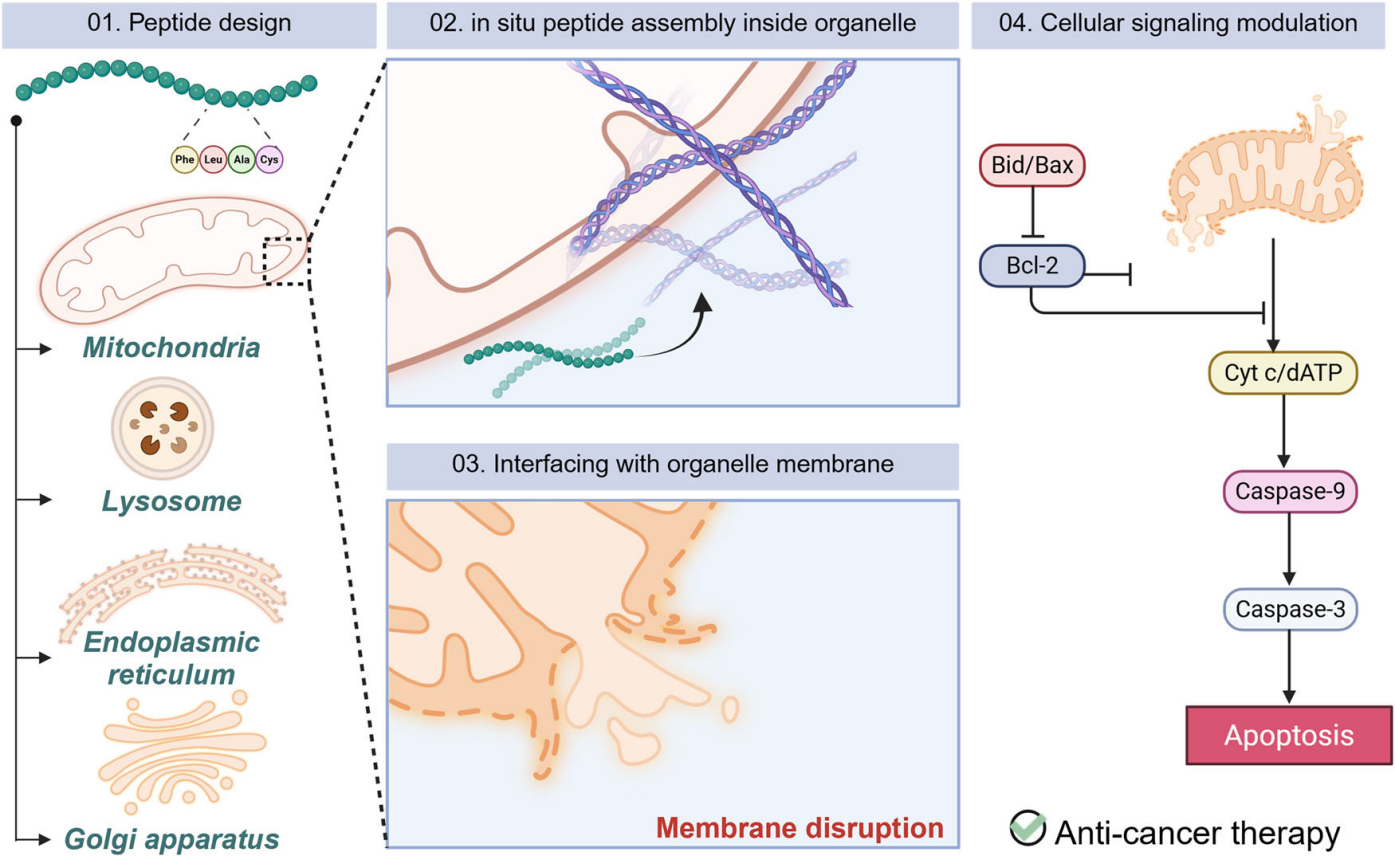
This schematic illustration represents biomimetic peptide self-assembly interfacing with organelle membranes. Peptide building blocks are rationally designed according to the characteristics of each target organelle. In response to organelle-specific stimuli, in situ peptide self-assembly occurs within the organelle, producing nanostructures that interact with and disrupt the membrane. These membrane–assembly interactions modulate intracellular signaling pathways, ultimately leading to therapeutic effects such as anticancer activity. The figure was created with BioRender.com.

The dynamic nature of the lipid bilayer provides a fluid yet selectively permeable matrix capable of regulating the transport of ions, metabolites, and signaling molecules across cellular compartments. Embedded or peripheral receptors within the bilayer mediate multivalent ligand–receptor interactions that trigger intracellular signaling cascades^42,43^. Such cooperative interactions across the membrane interface are central to cell communication, homeostasis, and adaptation to external stimuli. Given its structural and functional significance, the lipid bilayer is now widely recognized as a crucial platform for designing biomimetic peptide assemblies that can interface with biological membranes. Artificial peptide self-assemblies can be engineered to form on or within lipid bilayers, enabling dynamic interactions that modulate cellular signaling pathways, membrane curvature, or disruption. This emerging strategy provides a powerful approach to reprogram biological responses through supramolecular chemistry at the membrane interface (Fig. 2). In the following sections, we discuss how peptide self-assemblies formed on lipid bilayers of mitochondria, ER, lysosomes, and Golgi apparatus influence distinct cellular signaling processes (Table 1).

**Table 1:** Summary of intracellular peptide assembly interfacing with organelle membrane-mediated cell function modulation.

Name	Target sequence	Location	Function	Ref.
Mito-FF	Py-FFK-TPP	Mitochondria	Cytochrome *c* release → apoptosis	^46^
Heterochiral Mito-FF	(L)-Mito-FF + (D)-Mito-FF			^49^
Mito-SA	Succinic anhydride-modified Mito-FF			^50^
RC-K-FX	KF_x_AKF_x_AK		NF-κB suppression → apoptosis	^51^
MNP	RRF_x_RF_x_R		ATP sequestration → apoptosis	^52^
Mito-K2	KLAKLAKRGD		p53 activation → apoptosis	^53^
Fc-TPP1	Fc-DWpDYDFGDK-TPP		Ferroptosis and apoptosis	^54^
Pep-AT	Py-FFK(acetazolamide)K(TPP)	Lysosome	LMP → apoptosis	^58^
Benz-A_6_-RGD	Benzyl-AAAAAARGD		LMP → HSP70 downregulation → cathepsin B release → apoptosis	^59^
Py-Tyr	Py-Y_p_		LMP → cathepsin B release → apoptosis	^60^
NDI-Lyso-RGD	NDI-FFRRRRKRGD		LMP → apoptosis	^61^
Py-Y_p_-Lyso	Py-FFEY_p_		Caspase 3 activation → Bcl-2 downregulation	^62^
AmpF	FF-Amp-FF		Lysosomal endocytosis and subsequent lysosomal escape	^63^
KH-15	KKKHWLLLLLLH-C6-HHH			^64^
1P	Nap-FFY_p_R	Endoplasmic reticulum	BiP/CHOP upregulation → PERK-eIF2α activation → apoptosis	^66^
C_6_RVRRF_4_KY	CCCCCCRVRRFFFFKY	Golgi apparatus	GBF1/GALT1 reduction → BAX translocation → cytochrome *c* release → apoptosis/necrosis	^69^
NF-1	RGDRVRRKLVFF		MIF downregulation → immune activation	^70^
pS1	NBD-FF-thiophosphate		Oncosis	^71^

NF-κB, nuclear factor kappa-light-chain-enhancer of activated B cells. *ATP* adenosine triphosphate, *LMP* lysosomal membrane permeabilization, *HSP70*, heat shock protein 70, *Bcl-2*, B-cell lymphoma 2, *BAX*, BCL2-associated X protein, *BiP* binding immunoglobulin protein, *CHOP*, CCAAT/enhancer-binding protein homologous protein, *PERK* protein kinase RNA activated-like endoplasmic reticulum kinase, *eIF2α* alpha subunit of eukaryotic initiation factor-2, *GBF1* golgi brefeldin A resistance factor 1, *GALT1* galactosyltransferase 1, *MIF* migration inhibitory factor.

### Mitochondrial membrane

The mitochondria are a central organelle governing cellular energy metabolism, redox balance, and apoptosis through its double-membrane structure. Disruption of its membrane integrity readily collapses the electrochemical potential (ΔΨ_m_), releases cytochrome *c*, and activates cell-death signaling^44,45^. However, the dense double membrane and strong negative potential make mitochondria a challenging yet attractive target for molecular intervention. To achieve selective mitochondrial localization, peptide self-assembly systems are often engineered with mitochondria-targeting ligands or with mitochondria-affinitive sequences. By coupling these targeting elements to self-assembling backbones, the peptides remain monomeric in the cytoplasm but assemble in situ on the mitochondrial membrane, where the resulting nanostructures can perturb the mitochondria membrane, affecting mitochondria-related cellular signaling.

**Fig. 3:**
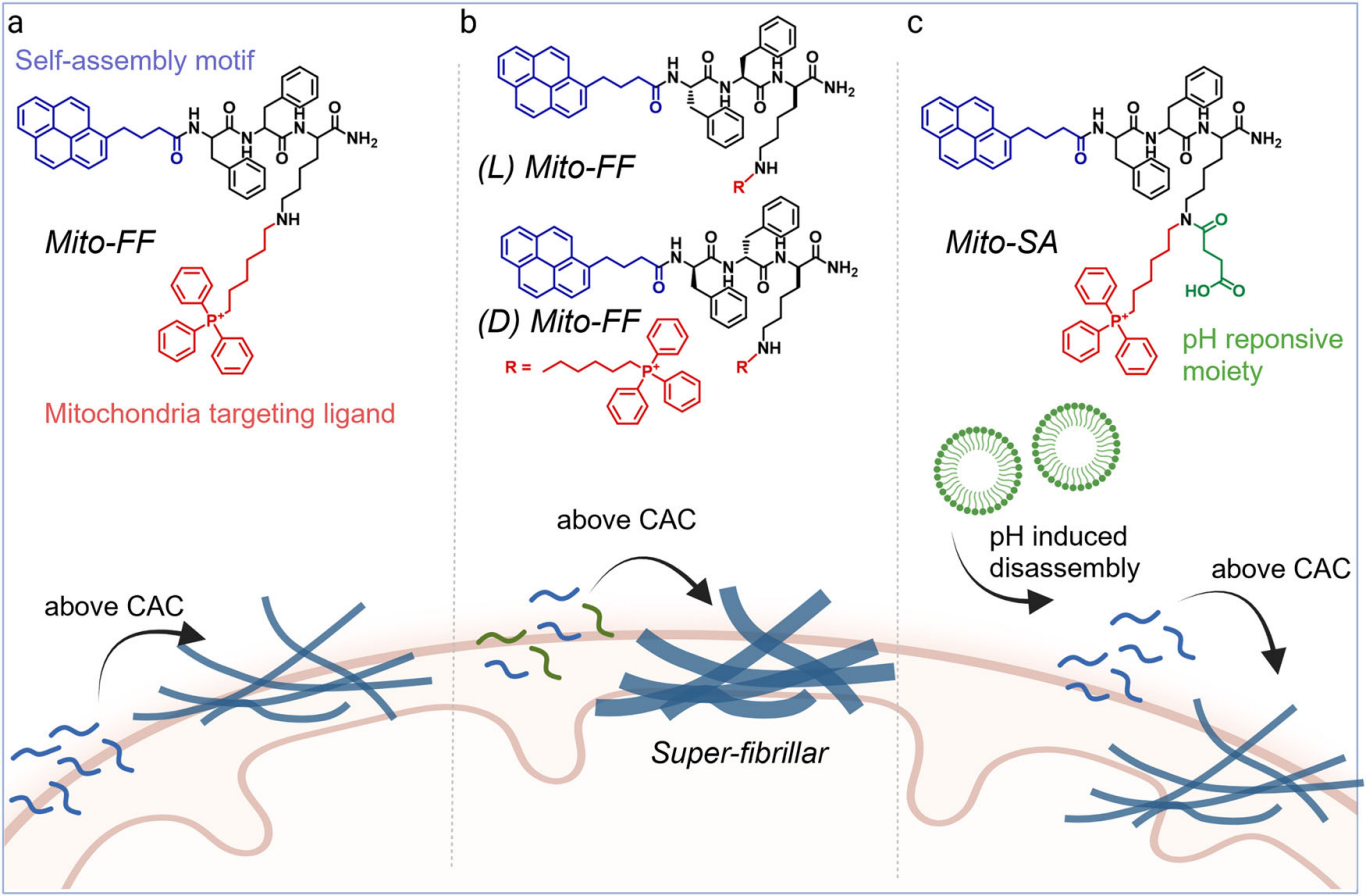
Schematic illustration showing the chemical structures of Mito-FF and Mito-SA, and the subsequent intracellular peptide self-assembly process. **a** Molecular structure of Mito-FF, containing a mitochondria targeting ligand. Mito-FF self-assembles on the mitochondrial membrane when its concentration exceeds CAC. **b** Monomer structures of the L- and D-forms of Mito-FF. These two monomers co-assemble on mitochondrial membrane to form supramolecular super-fiber. **c** Molecular structure of Mito-SA, which contains a pH-responsive moitey. Under disease-associated acidic conditions, Mito-SA is transformed into Mito-FF, leading to mitocnodrial self-assembly. CAC critical aggregation concentration. The figure was created with BioRender.com.

A representative example of peptide self-assembly interacting with the mitochondrial membrane is Mito-FF, which consists of a pyrene-conjugated diphenylalanine (Py-FF) backbone linked to a mitochondria-targeting ligand, triphenylphosphonium (TPP)^46–48^ (Fig. 3a). In the cytoplasm, the molecule remains molecularly dispersed because its concentration is below the critical aggregation concentration. However, the strong negative potential of the mitochondrial membrane drives TPP-mediated accumulation, enabling the peptide to exceed its critical aggregation concentration and undergo in situ self-assembly inside mitochondria. The resulting nanofibrillar assemblies intimately interact with the lipid bilayer, inducing membrane disruption and protein leakage, which ultimately collapse the mitochondrial membrane potential and activate intrinsic apoptotic signaling. Structural modulation of this backbone further enhances functional control. When the L-form of Mito-FF is co-assembled with its D-form enantiomer, the heterochiral mixture forms super-fibrillar structures inside mitochondria with accelerated assembly kinetics, increased mechanical rigidity, and improved enzymatic stability compared with homochiral analog^49^ (Fig. 3b). These mechanically robust super-fibrils produce a mitochondrial stress response and potent apoptotic induction. In addition, chemical modification of the Mito-FF primary amine with succinic anhydride generates a transformable, tumor-microenvironment (TME)-responsive precursor^50^ (Fig. 3c). Under acidic TME conditions, the anhydride group is cleaved, restoring the cationic character of TPP and triggering mitochondrial accumulation followed by self-assembly inside mitochondria. The activated nanostructures subsequently disrupt the mitochondrial membrane, promoting cytochrome *c* release and cell death.

Beyond small-molecule ligand conjugation, peptide self-assembly-interfacing mitochondrial membrane can also be achieved through the rational design of peptide sequences that inherently possess mitochondrial affinity. Among them, hydrophobic-cationic repeating sequences, such as KF_x_AKF_x_AK, have proven particularly effective in mediating self-assembly inside mitochondria, resulting in membrane interaction^51^. These peptides combine amphiphilic residues and lysine-mediated electrostatic attraction toward the negatively charged mitochondrial membrane. Once internalized, they undergo in situ self-assembly inside mitochondria, forming nanofibrillar aggregates that strongly interact with both the outer and inner mitochondrial membranes. Such membrane engagement induces structural disruption, leading to the loss of membrane potential and leakage of mitochondrial proteins. Consequently, these events trigger intrinsic apoptotic cascades and markedly amplify oxidative stress within the cell. The resulting oxidative burst further suppresses the nuclear factor-κB signaling pathway, a key transcriptional regulator of cell survival and inflammation, thereby reducing anti-apoptotic gene expression and reinforcing apoptosis progression. Building upon this concept, hydrophobic–cationic repeating sequences have been further diversified to include nucleopeptide variants capable of binding mitochondrial ATP through complementary base-pairing interactions. Specifically, the adenine nucleobase of ATP can form a strong Watson–Crick base pair with thymine, providing a non-covalent recognition motif for ATP sequestration. Guided by this principle, an RRF_x_RF_x_R modified with nucleopeptide (MNP) was developed, consisting of a thymine nucleobase for association with mitochondrial ATP^52^. Initially, MNP electrostatically associates with ADP to form small aggregated particles (MNP/ADP). MNP/ADP complex accumulates within mitochondria through its intrinsic hydrophobic-cationic repeated sequence, leading to the formation of large supramolecular micellar assemblies by selectively capturing ATP over ADP within the mitochondria. These assemblies intimately engage mitochondrial membrane, sequestering ATP and perturbing mitochondrial energy metabolism. These processes induce membrane depolarization, reactive oxygen species (ROS) accumulation, and cytochrome *c* release, which collectively activate caspase-dependent apoptotic signaling.

In another variant designed for pathological selectivity, a dithiol-linked KLAKLAKRGD peptide integrates both a mitochondrial-targeting motif (KLAKLAK) and a redox-sensitive disulfide bridge that responds to elevated ROS levels in senescent cells^53^. Under high ROS conditions, the dithiol bond undergoes oxidation and cleavage, triggering in situ oligomerization within mitochondria. The resulting supramolecular assemblies permeabilize the mitochondrial membrane, causing depolarization, cytochrome *c* efflux, and caspase 9/3 activation. Concurrently, these assemblies enhance p53-mediated senolytic responses, selectively inducing apoptosis in aging while sparing normal tissues.

In addition to nanofibrillar assemblies, mitochondrial membrane interaction can also be achieved through non-fibrous supramolecular morphologies. For example, a ferrocenyl-modified, mitochondria-targeting peptide (Fc-TPP1) was engineered to form a host–guest regulated, enzyme-instructed self-assembly system that generates supramolecular nanospheres^54^. By using pillar[6]arene to kinetically modulate enzymatic self-assembly, the peptide remains dispersed during cellular uptake and subsequently reassembles into nanospheres inside mitochondria following lysosomal escape. These mitochondria-localized nanospheres intimately interact with mitochondrial membranes, induce membrane depolarization, promote ROS accumulation, and trigger ferroptosis-associated and apoptotic signaling pathways, ultimately leading to selective cancer cell death.

Collectively, these peptide sequence-driven mitochondrial assemblies demonstrate how amino acid motifs can be programmed to perform complex biological functions through spatiotemporally controlled self-assembly at the organelle membrane interface. By coupling mitochondrial targeting elements with self-assembling backbones, peptides can remain monomeric in the cytoplasm yet form supramolecular nanostructures within mitochondria, where they disrupt membrane integrity and remodel mitochondrial signaling. These assemblies directly influence key pathways, such as ROS regulation, NF-κB suppression, p53 activation, ferroptosis, and caspase-mediated apoptosis, demonstrating how supramolecular dynamics can translate molecular interactions into defined cellular outcomes. Additionally, their ability to sense mitochondrial biochemical cues, including membrane potential, ATP concentration, and oxidative stress, and to adaptively transform into functional assemblies positions them as synthetic analogs of biological signaling machinery. Continued integration of enzyme-responsive, redox-responsive, or nucleic acid-responsive motifs could further refine their precision, opening new opportunities to reprogram mitochondrial metabolism. Altogether, these advances highlight the potential of bioinspired supramolecular peptide systems as versatile tools for modulating mitochondrial function and developing next-generation therapeutics.

### Lysosomal membrane

The lysosome is an acidic, enzyme-rich organelle that serves as the terminal compartment of the endocytic and autophagic pathways, responsible for degrading macromolecules and maintaining cellular homeostasis^55–57^. Beyond its degradative role, the lysosome also acts as a crucial signaling platform that regulates metabolism, autophagy, and programmed cell death. The integrity of the lysosomal membrane is essential for cell survival; disruption of this barrier, known as lysosomal membrane permeabilization (LMP), leads to the release of cathepsins and other hydrolases into the cytosol, initiating apoptotic or necrotic pathways. Owing to its distinct microenvironment (pH ~ 4.5 and abundant hydrolytic enzymes), the lysosome provides a unique niche for the design of stimuli-activatable peptide assemblies that are dormant under neutral conditions but become self-assembling and functional once internalized into the lysosomal lumen. To construct peptide assemblies that are activated specifically within lysosomes, two major approaches have been established: (i) pH-triggered assembly exploiting the acidic milieu and (ii) enzyme-instructed assembly responsive to lysosomal proteases such as cathepsin B or glucuronidase.

**Fig. 4:**
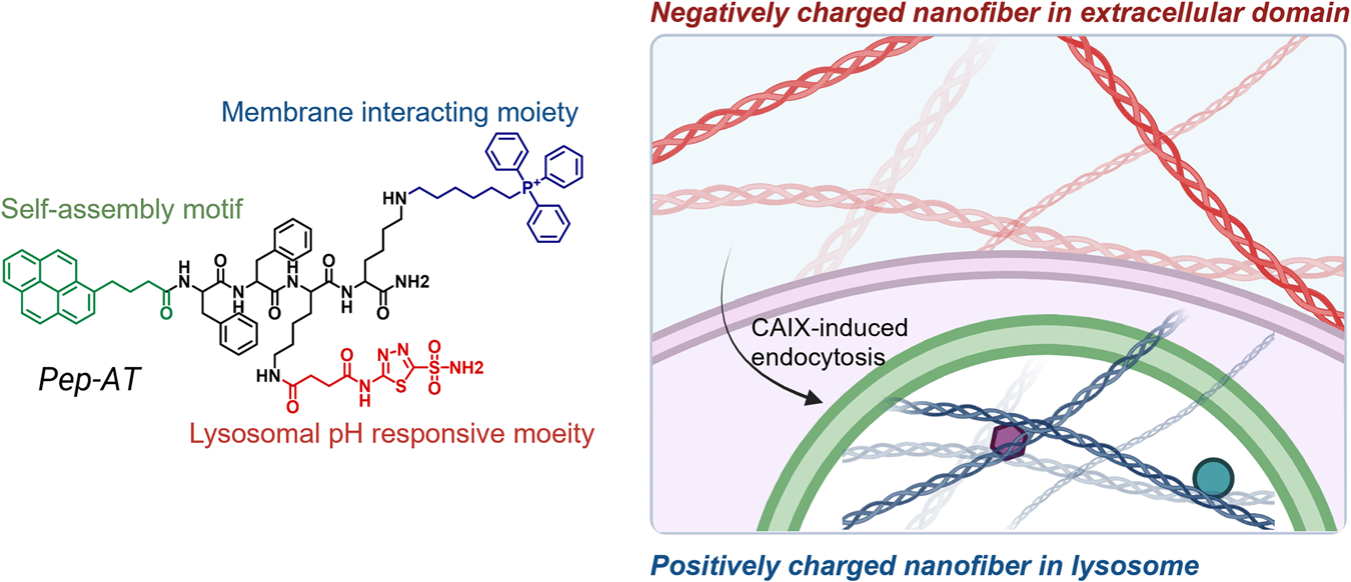
Chemical structure and self-assembly behavior of Pep-AT. Initially, Pep-AT forms negatively charged nanofibers in the extracellular environment, which are subsequently internalized into lysosomes via carbonic anhydrase IX (CAIX)-mediated endocytosis. Inside the lysosome, the acidic pH environment induces a charge reversal, transforming the negatively charged nanofibers into positively charged ones that interface with and disrupt the lysosomal membrane. The figure was created with BioRender.com.

pH-responsive peptide monomers are designed to remain disassembled at physiological pH (7.4) but undergo protonation and reduced electrostatic repulsion under acidic lysosomal conditions, leading to spontaneous aggregation and nanofiber formation. A representative system is the pyrene-conjugated FF dipeptide functionalized with TPP and acetazolamide^58^ (Pep-AT, Fig. 4). At neutral pH, acetazolamide exists in a negatively charged form, maintaining the peptide in an inactive state. However, after carbonic anhydrase IX-mediated endocytosis into lysosomes, the pH-dependent neutralization enhances the positive charge of TPP, which in turn induces LMP and activates downstream apoptotic signaling. Similarly, the amphiphilic oligopeptide Benz-AAAAAARGD forms non-toxic peptosomes under neutral conditions but transforms into elongated nanofibers at pH 4.5 (ref. ^59^). These nanofibers disrupt the lysosomal membrane, inhibit the expression of the cytoprotective protein HSP70, and trigger cathepsin B release, collectively leading to apoptotic cell death. A simpler pH-responsive assembly is illustrated by pyrene-capped tyrosine (Py-Tyr), which self-assembles into micron-sized aggregates under acidic conditions through protonation-induced charge neutralization^60^. The resulting large assemblies exceed the typical size of lysosomes, causing lysosomal rupture and the release of cathepsin B, which further amplifies apoptotic signaling cascades.

In another approach, enzyme-responsive peptide assemblies utilize lysosomal proteases to control activation. The NDI-conjugated FFRRRRKRGD peptide exemplifies this concept: guided by the RGD motif, it accumulates in tumor lysosomes, where cathepsin B cleaves the RRRRK sequence^61^. The proteolytic event converts ~30 nm nanomicelles into large nanofibers (>1 μm), which destabilize the lysosomal membrane and initiate apoptosis. Structural variants of these peptide-bearing different N-terminal groups, such as amine, C16, or pyrene, exhibit correlated changes in self-assembly propensity and cytotoxicity, confirming that cathepsin B-mediated structural transformation governs lysosomal disruption. Another example involves a glycopeptide bearing glucuronic acid and glucose units, which undergoes β-glucuronidase-triggered self-assembly within lysosomes to form nanofibers that induce LMP, cytoskeletal disassembly, and apoptosis. Similarly, a morpholine-conjugated Py-Y_p_ peptide monomer, responsive to both alkaline phosphatase (ALP) on the plasma membrane and the acidic lysosomal environment, can form nanofibers that translocate into lysosomes and disrupt their membranes^62^. This ALP/lysosome dual-responsive system not only facilitates endosomal–lysosomal escape but also alters intracellular signaling by activating caspase 3, downregulating Bcl-2, and promoting cathepsin-dependent apoptosis.

In addition, peptide-based supramolecular self-assembly can be exploited to regulate lysosome-involved cellular trafficking. A representative example is a TME adaptable self-assembly system based on a short pentapeptide containing a pH-sensitive 4-amino-proline residue (AmpF)^63^. Under neutral extracellular conditions, AmpF co-assembles with a photosensitizer-conjugated analog (AmpF-C) into elongated superhelical nanostructures, which favor prolonged circulation and tumor accumulation. Upon cellular uptake via endocytosis, the assemblies are trafficked into endosomal/lysosomal compartments, in which the acidic environment (pH ~5.5) induces *cis–trans* isomerization of the Amp amide bond, triggering a morphological transition into spherical nanoparticles. Notably, prolonged incubation led to a progressive decrease in lysosomal colocalization, indicating efficient lysosomal escape of the nanoparticles into the cytosol. In another example, efficient lysosomal escape is provided by de novo designed pH-responsive peptide nanoparticles that enable cytosolic delivery of nucleic acid^64^. In this system, short amphiphilic peptides were rationally engineered by modularly integrating hydrophobic leucine segments, pH-responsive histidine networks, and lysine-rich cationic domains for nucleic acid binding. Following endocytosis of formed nanoparticles, they traffic through early endosomes and subsequently late endosomes/lysosomes. Crucially, protonation of histidine residues at endosomal pH induces a cooperative destabilization of the supramolecular assembly, leading to increased surface charge, membrane interaction, and selective disruption of endosomal and lysosomal membranes. By precisely tuning the transition pH to match specific endosomal compartments, the timing and efficiency of lysosomal escape can be controlled. Collectively, this system demonstrates how peptide-based nanoparticles can exploit lysosomal endocytosis and subsequent lysosomal escape as a programmed activation route for intracellular signaling.

Collectively, these examples underscore how lysosome-activated peptide assemblies transform a degradative organelle into a controllable supramolecular reaction hub. By integrating environmental cues, such as pH gradients and lysosomal enzyme activity, into molecular design, peptides can undergo spatiotemporally regulated self-assembly within lysosomes, converting inert precursors into functionally active nanostructures. Once assembled, these supramolecular architectures exert various roles. First, it can physically perturb the lysosomal membrane through LMP induction and biochemically reprogramming downstream signaling pathways. The release of cathepsins, caspase activation, and modulation of apoptosis-regulating proteins such as Bcl-2 and HSP70 illustrate how the structural dynamics of these assemblies are directly coupled to regulate cellular signaling. Second, it can harness lysosomal endocytosis and subsequent lysosomal escape to program intracellular signaling activation. Future designs that combine multistimuli responsiveness (pH, redox, and enzyme cues) or integrate targeting ligands for specific lysosomal pathways may enable selective degradation of pathological cells while sparing normal tissues. Such strategies could be leveraged not only for cancer therapy but also for diseases associated with lysosomal dysfunction, including neurodegenerative and lysosomal storage disorders. Altogether, lysosome-activated peptide self-assembly provides a paradigm for dynamic molecular therapeutics, in which structural adaptability and organelle selectivity converge to achieve precision control over intracellular signaling and cell fate.

### Endoplasmic reticulum membrane

The ER is a multifunctional organelle responsible for protein folding, calcium storage, and lipid biosynthesis, serving as a central node in cellular signaling and homeostasis^65^. Although ER is an attractive therapeutic candidate, the ER remains a particularly challenging organelle to target selectively. Its dynamic membrane sites and morphological plasticity make it difficult for most supramolecular systems to distinguish the ER from neighboring compartments. Consequently, only a few molecular designs have successfully achieved selective activation and self-assembly on the ER membrane.

An enzyme-instructed aromatic peptide amphiphile, consisting of naphthyl-diphenylalanine-lysine-acetazolamide (Nap-FFK-acetazolamide), has been demonstrated^66^. Upon phosphatase-catalyzed dephosphorylation, the peptides transform into a hydrophobic amphiphile that spontaneously organizes into crescent-shaped supramolecular assemblies. These assemblies preferentially interact with the ER membrane, where their insertion and aggregation disrupt the lipid packing and induce localized ER stress. The disruption activates the unfolded protein response, elevating the expression of BiP (GRP78) and CHOP, two canonical ER-stress markers associated with the PERK–eIF2α pathway. Sustained activation of this stress pathway shifts the unfolded protein response from a protective mode to a pro-apoptotic mode, ultimately resulting in irreversible ER stress and programmed cell death. This example illustrates how enzymatically triggered amphiphilic peptides can harness the intrinsic sensitivity of the ER membrane to create self-amplifying feedback between supramolecular organization and stress signaling.

ER-responsive peptides reveal that subtle control over molecular design can dictate how assemblies interface with ER membranes. Their activation translates structural transformation into defined biological consequences, including ER stress and apoptosis. By coupling the chemical programmability of peptide self-assembly with the intrinsic signaling plasticity of ER, such systems offer a powerful route to manipulate organelle-specific stress pathways and to explore the therapeutic potential of supramolecular chemistry within the endomembrane network.

### Golgi apparatus

The Golgi apparatus has a pivotal role in the protein signaling pathway, functioning as a central hub for trafficking, sorting, and post-translational modification of biomolecules^67,68^. Structurally composed of flattened cisternal stacks, the Golgi mediates the directional transport of proteins and lipids between the ER, plasma membrane, and other intracellular compartments. Beyond its classical role in vesicular trafficking, the Golgi has emerged as a key signaling platform that integrates and redistributes various molecular cues, thereby coordinating cellular processes such as secretion, migration, proliferation, and stress responses. Owing to its central role in intracellular signaling and molecular trafficking, increasing attention has been directed toward constructing peptide-based supramolecular assemblies on the Golgi membrane to modulate a signaling pathway. By interacting with the Golgi membrane, such assemblies can alter local microenvironments and influence Golgi-mediated signaling cascades. To achieve the selective formation of activated self-assembled structures within the Golgi apparatus, researchers have widely utilized the enzyme Furin, which is highly expressed in the Golgi membrane. Furin specifically recognizes and cleaves the peptide sequence RVRR, providing a biochemical trigger for in situ activation. This strategy allows the design of inactive precursors that remain non-assembling or non-toxic until enzymatic cleavage, after which active peptide nanoassemblies are generated within the Golgi environment.

For example, the CCCCCCRVRRFFFFKY initially forms non-toxic nanoparticles in aqueous solution^69^. Upon cellular uptake and subsequent Furin-mediated cleavage inside the Golgi, these peptides undergo structural transformation into left-handed helical fibrils. The resulting fibrillar assemblies physically disrupt the Golgi membrane, leading to a marked reduction in key Golgi-associated proteins such as guanine nucleotide exchange factor 1 and galactosyltransferase 1. This membrane perturbation further triggers a cascade of apoptotic signaling events, including the translocation of pro-apoptotic BAX from the cytosol to the mitochondria and the concomitant release of cytochrome *c* into the cytosol. In parallel, a global decrease in cytokine expression is observed, collectively driving both apoptotic and necrotic cell death. A similar concept has been demonstrated with another Golgi-targeting self-assembling peptide, NF-1 (RGDRVRRKLVFF)^70^. In this system, the peptide remains in a non-toxic, unassembled state before enzymatic cleavage, but reassembles into cytotoxic nanostructures upon Furin activation. When administered to tumor cells, the NF-1 assemblies disrupt the Golgi membrane and downregulate tumor cell-derived GA-dependent migration inhibitory factor, a key mediator of the immunosuppressive network within cancer cells. Consequently, this process reshapes the tumor immune microenvironment, converting a “cold” tumor into a “hot” tumor through enhanced immune signaling and activation.

Beyond Furin-responsive systems, alternative enzyme-instructed strategies have also been developed to induce peptide self-assembly specifically on the Golgi membrane^71,72^. NBD-terminated diphenylalanine (FF) thiophosphate peptide (pS2) was designed as an ALP cleavable precursor. Initially, pS2 exists as a soluble monomeric species; however, upon internalization and ALP-mediated dephosphorylation, it undergoes in situ assembly on the Golgi membrane, leading to the formation of fluorescent nanostructures. Interestingly, structural tuning through naphthyl substitution on the aromatic motif further enhanced Golgi localization and promoted membrane oxidation, which contributes to cytotoxicity. The ALP-triggered Golgi oxidation not only perturbs the membrane integrity but also initiates a unique form of regulated cell death distinct from classical apoptosis or necroptosis. In line with this, several conventional inhibitors of apoptosis and ferroptosis (Z-VAD-FMK, *N*-acetylcysteine, Nec-1, deferoxamine, ferrostatin-1, and disulfiram) failed to rescue HeLa cells from pS2-induced cytotoxicity. By contrast, treatment with the oncosis inhibitor PD150606 partially restored cell viability, suggesting that Golgi membrane-associated oxidative oncosis is the dominant death pathway triggered by this ALP-activated self-assembly system.

Collectively, these studies reveal that enzyme-activated peptide assemblies on the Golgi membrane offer an effective strategy to reprogram organelle function through precisely controlled supramolecular organization. By exploiting Golgi-localized enzymes such as Furin or ALP, otherwise inert peptides can undergo in situ structural transformation within the Golgi lumen, converting soluble precursors into functionally active nanostructures. These assemblies physically perturb the Golgi membrane, impairing vesicular trafficking and post-translational processing, while activating stress-related and apoptosis-related pathways that extend to other organelles such as the ER and mitochondria. This interplay highlights the Golgi’s role not only as a trafficking center but also as a signaling node in which supramolecular transformation can initiate cascading biological consequences across the cell. Furthermore, the ability to trigger distinct cell-death modalities from classical apoptosis to oxidative oncosis demonstrates how subtle changes in assembly kinetics, molecular packing, or enzymatic responsiveness can dictate specific cellular outcomes.

## Biomimetic peptide self-assembly interfacing with proteins to regulate cellular signaling

Proteins are the most abundant functional macromolecules in living cells, distributed across distinct compartments such as the plasma membrane, cytosol, mitochondria, and nucleus. Through finely tuned PPIs, they orchestrate dynamic signaling networks that regulate metabolism, proliferation, apoptosis, and immune homeostasis. Membrane proteins — receptors, channels, and transporters — mediate extracellular communication and environmental sensing, whereas cytosolic kinases and adaptor proteins propagate intracellular signals to coordinate growth or programmed cell death. Mitochondrial enzymes and channel proteins link metabolic flux to cell survival, and nuclear transcription factors shape gene-expression programs underlying differentiation and stress adaptation. Because cellular functions rely on the spatial and temporal organization of these PPIs, their dysregulation often triggers pathophysiological processes such as cancer progression, inflammation, or metabolic imbalance.

To reprogram such aberrant protein networks, biomimetic peptide self-assemblies have emerged as powerful tools capable of interfacing with proteins through well-defined supramolecular architectures. Unlike conventional small-molecule inhibitors that rely on discrete catalytic pockets, peptide assemblies mimic extended protein–protein interfaces by presenting secondary structural motifs — α-helices, β-sheets, coiled-coils, or amphiphilic nanofibers — that recapitulate the geometry and chemical environment of native binding domains. This hierarchical organization enables multivalent recognition, cooperative binding, and high structural complementarity across broad, shallow surfaces previously considered “undruggable.”^73–75^ By precisely positioning functional residues, peptide assemblies can competitively block or stabilize PPIs, induce receptor clustering or degradation, and spatially reorganize signaling complexes within specific subcellular locations.

**Fig. 5:**
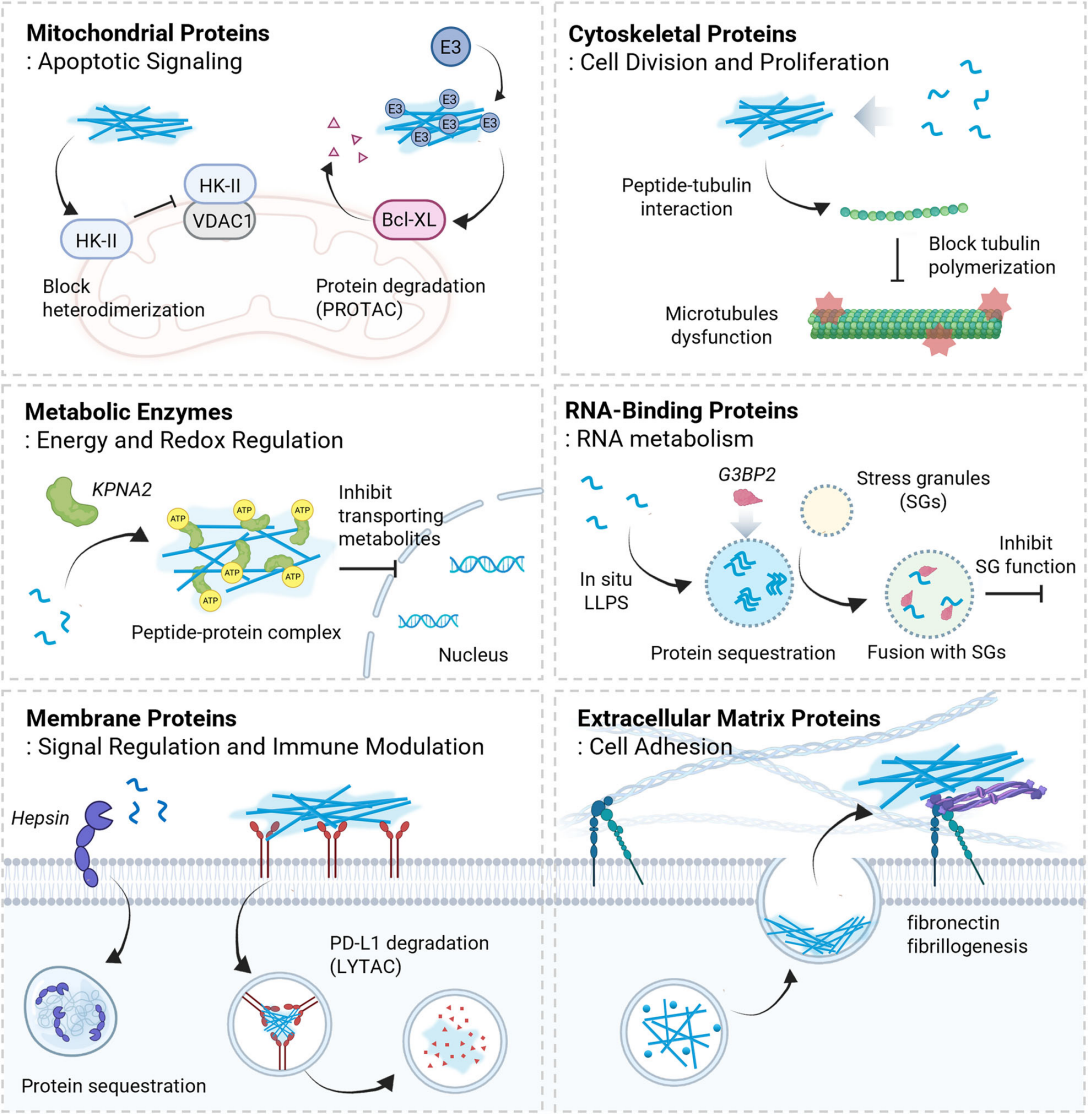
This schematic illustration represents interfacing peptide with proteins modulating cellular function. Peptide assemblies interfacing with diverse classes of cellular proteins, including mitochondrial or cytoskeletal proteins involved in cellular signaling processes such as division, proliferation, and apoptosis: metabolic enzymes responsible for energy and redox regulation; RNA-binding proteins associated with RNA metabolism; and membrane or extracellular matrix proteins that regulate signaling, immune modulation, or cell adhesion. HK-II hexokinase II, VDAC1 voltage-dependent anion channel 1, Bcl-XL B-cell lymphoma-extra large, KPNA2 karyopherin subunit alpha-2, G3BP2 Ras-GAP SH3 domain-binding proteins 2, LLPS liquid–liquid phase separation, SGs stress granules, PD-L1 programmed death-ligand 1. PROTAC proteolysis-targeting chimera. LYTAC lysosome-targeting chimera. The figure was created with BioRender.com.

Through this interface-driven modulation, peptide assemblies provide a modular means to regulate diverse protein classes involved in cellular signaling. Depending on their localization and molecular targets, such systems have been applied to (i) mitochondrial proteins governing apoptotic cascades, (ii) cytoskeletal proteins controlling cell division and motility, (iii) metabolic enzymes modulating redox and energy balance, (iv) membrane proteins engaged in immune or receptor signaling, and (v) extracellular matrix (ECM) proteins mediating adhesion and mechanotransduction. In the following sections, representative examples of these categories are discussed to illustrate how structural mimicry and dynamic assembly behavior of peptides converge to regulate protein activity and cellular fate (Fig. 5).

### Mitochondrial proteins involved in apoptotic signaling

The mitochondrial outer membrane (MOM) hosts a repertoire of regulatory proteins that orchestrate the initiation of apoptosis by controlling membrane permeability and the release of apoptogenic factors. Among them, the *Bcl-2* family proteins act as molecular switches between survival and death: anti-apoptotic members such as Bcl-xL and Bcl-2 sequester pro-apoptotic partners (Bax and Bak) to preserve mitochondrial integrity. Peptide assemblies have emerged as a powerful means to interfere with these PPIs, thereby reprogramming apoptotic signaling. For instance, assemblies incorporating the BH3 domain (GQVGRQLAIIGDDINR), such as Py^Me^Amp-BH3, competitively bind to the hydrophobic groove of Bcl-xL, displacing Bax and destabilizing the MOM to trigger cytochrome *c* release^76^. Furthermore, enzyme-responsive dual-ligand assemblies (Supra-PROTACs) use a bola-amphiphilic hexapeptide (EIYsIYsE) conjugated with both a BH3 domain and the VHL-recognition sequence (LAHypY1) from hypoxia-inducible factor-1α, enabling VHL recruitment and Bcl-xL degradation through ubiquitination^77^. In parallel, the HK-II–VDAC1 complex has a crucial role in maintaining cellular survival by coupling glycolytic ATP production to mitochondrial energy regulation^78^. Amphiphilic peptides are designed from the HK-II-binding fragment of VDAC1 (LP1, WTEYGLTFTEKWNTDN) and are modified with a hydrophobic N-terminal segment (palmitic acid or pFL) and a cationic C-terminal tail (TAT or RVR) to promote nanofibrous supramolecular assembly. These assemblies are competitively associated with HK-II, displacing its binding to VDAC1 and facilitating the detachment of HK-II from MOM^79^. These approaches illustrate how assembly-mediated modulation of mitochondrial PPIs can reprogram apoptotic signaling.

### Cytoskeletal proteins involved in cell division and proliferation

Cell division and proliferation rely on the dynamic organization of the cytoskeletal network, primarily composed of actin, tubulin, and intermediate filaments, whose polymerization and depolymerization are governed by highly coordinated PPIs. Disruption of these interactions can halt mitosis and trigger apoptosis, making cytoskeletal proteins attractive targets for peptide assembly-based intervention. For instance, a tyrosinase-activated tripeptide (FFY) self-assembles into diquinone-linked aggregates inside melanoma cells, where it interferes with tubulin–tubulin polymerization, leading to microtubule disassembly and G2/M arrest^80^. Likewise, Py-FFLLK (PyMT) peptides form intracellular assemblies upon release from folic acid-functionalized β-cyclodextrin, which interacts with microtubules and induces apoptotic collapse^81^. These examples highlight how interfacial peptide assemblies can precisely reprogram cytoskeletal dynamics, transforming structural scaffolds of proliferation into programmable targets for cancer therapy^82,83^.

### Metabolic enzymes involved in energy and redox regulation

Metabolic proteins act as key mediators of cellular homeostasis by regulating the translocation and conversion of essential metabolites such as ATP, glucose, and lactate. When these transport-associated interactions are perturbed, metabolic flux is disrupted, leading to energetic stress and cell death. Interfacing peptide assemblies exploit this vulnerability by engaging with metabolic enzymes to induce structural or functional blockage. For instance, transformable nucleopeptides (NLS-FF-T) interact with the nuclear transport protein *Karyopherin subunit alpha-2* (KPNA2), forming fibrous protein–peptide assemblies that expose ATP-binding sites and sequester intracellular ATP, resulting in mitochondriopathy-like energy depletion^84^. In addition to energy regulation, redox metabolism represents another critical axis of metabolic control. The antioxidant enzyme glutathione peroxidase 4 (GPX4) prevents ferroptosis by reducing lipid hydroperoxides in mitochondria. To block this protective function, a peptide–ferriporphyrin conjugate (Gi-F-CAA) was designed to self-assemble under acidic tumor conditions, generating nanoparticles (Gi-F) that bind to GPX4 with high affinity through the assembly-enhanced binding effect. This strong PPI inhibits GPX4 activity, elevates lipid peroxidation, and triggers ferroptotic cell death in tumor cells^85^.

### RNA-binding proteins involved in RNA metabolism

RNA-binding proteins are responsible for post-transcriptional gene regulation by controlling RNA localization, stability, translation, and stress-responsive reprogramming. Under cellular stress, many RNA-binding proteins dynamically reorganize into biomolecular condensates such as stress granules through liquid–liquid phase separation (LLPS), enabling rapid and reversible modulation of RNA metabolism. Inspired by these adaptive cellular mechanisms, recent studies have increasingly sought to mimic biomolecular condensates using peptide assemblies to induce intracellular LLPS and modulate biological activity through targeted interactions with RNA-binding proteins. For example, the peptide (FGDF-Y^SO4^F) undergoes in situ LLPS and sequester *Ras-GAP SH3 domain-binding proteins 2* (G3BP2), RNA-binding protein that has a critical role in stress granule formation. The association facilitates fusion with stress granules while inhibiting their function, thereby suppressing caspase-dependent apoptosis following stress-inducing drug administration^86^. This recent approach extends conventional rigid and highly ordered peptide assemblies toward soft, deformable, and fusion-prone structures, resembling dynamic intracellular condensates.

### Membrane proteins involved in signal regulation and immune modulation

Membrane proteins have central roles in transducing extracellular cues into intracellular responses, governing cell adhesion, communication, and immune recognition. Interfacing peptide assemblies with these membrane proteins provides an effective means to reprogram receptor signaling, immune checkpoints, and enzymatic activities at the cell surface. For instance, *integrin α*_*2*_*β*_*1*,_ a transmembrane receptor anchoring cells to the ECM, can engage with pyrene-grafted amphiphilic peptides containing the Asp–Gly–Glu–Ala (DGEA) motif. These peptides self-assemble via π–π stacking into planar nanosheets with high stability and large surface area, and their continuous engagement with integrin α_2_β_1_ promotes myogenic proliferation and differentiation. This example highlights how engineered peptide assemblies interface with structural membrane proteins to regulate cell–matrix communication^87^.

Moreover, antibodies, as membrane-binding immune mediators, can be incorporated into peptide–antibody co-assemblies to construct hybrid supramolecular networks that modulate immune signaling. In the peptide–antibody combo assembly (PAC-SABI) system, enzyme-mediated amide coupling and multivalent electrostatic interactions produce in situ bi-targeting nanoarchitectures on cancer cell membranes that simultaneously block CD47 and CD24 checkpoints. This dual blockade restores macrophage recognition, promotes M1 polarization, and enhances antitumor immunity, exemplifying how peptide–protein co-assembly enables synchronized modulation of innate and adaptive immune responses^88^.

In parallel, the immune checkpoint protein PD-L1 serves as a target for lysosome-targeting chimera (LYTAC)-based assemblies. Self-assembling pyrene-capped peptides (Py-FFK-BMS) conjugated with a small-molecule PD-L1 ligand (BMS-8) form nanofibrous peptide–protein co-assemblies that anchor onto PD-L1-rich membranes. These supramolecular chimeras recruit lysosomal trafficking machinery, leading to PD-L1 internalization and degradation through catalytic lysosomal activity^89^. Likewise, epidermal growth factor receptor (EGFR), a transmembrane tyrosine kinase regulating cell proliferation and survival, can be functionally confined by cyclic peptide-based self-assembling scaffolds (PAD-1). The PAD-1 contains an EGFR ectodomain-binding motif (KLARLLT) and assemblies cluster EGFR through multivalent ectodomain binding, suppressing downstream signaling and inducing receptor internalization followed by lysosomal degradation and ER stress-mediated cell death^90^. As another immunotherapeutic strategy, in situ peptide assembly at the cell–cell interface can function as a molecular bridge connecting T cells and tumor cells. The dual-specific peptide anti-CD3-G7-RGD (anti-CD3–GNNQQNYRGD) simultaneously binds to CD3 receptors on T cells and integrin α_v_β_3_ on tumor cells, thereby bringing immune and cancer cells into close proximity. Upon dual recognition, the peptide undergoes conformational stabilization, which promotes localized peptide–protein co-assembly and receptor clustering at the membrane interface, ultimately inducing CD3 oligomerization and T cell activation^91^.

Emerging evidence suggests that peptide–protein assemblies can undergo condensation transitions at membrane interfaces. The amphiphilic-branched peptide (DMN-SIPL), integrating a hepsin-binding domain, forms ordered condensates through specific recognition with membrane-bound hepsin, a type II serine peptidase frequently overexpressed in prostate cancer. These condensates selectively disrupt hepsin-dependent signaling and metabolic pathways, leading to targeted growth inhibition in cancer cells^92^.

### Extracellular matrix proteins involved in cell adhesion

ECM proteins such as collagen and fibronectin have essential roles in cell adhesion, migration, and tissue organization through multivalent interactions with integrins and other membrane receptors. Interfacing peptide assemblies with these proteins provides a means to structurally modulate the cell–matrix interface and reprogram collective cellular behaviors^93,94^. For example, enzyme-responsive D-phosphopeptides (NBD-ffs_p_y) undergo endosomal dephosphorylation, forming helical nanofibers stabilized by hydrogen bonding and π–π stacking. Upon secretion, these nanofibers associate with fibronectin via hydrophobic interactions, promoting fibronectin fibrillogenesis and hybrid network formation. The resulting protein–peptide matrices act as artificial extracellular scaffolds, enhancing cell adhesion, spheroid formation, and tissue-like assembly^95^. In summary, interfacing peptide assemblies acts as tunable scaffolds that mediate ECM remodeling and receptor engagement, offering a biomimetic platform for controlling adhesion-driven cell organization.

In summary, this section has highlighted biomimetic peptide assemblies using proteins as targets for PPI modulation and integrating them as active structural components within self-assembling peptide networks. This cooperative assembly exemplifies how proteins can serve as structural organizers of peptide assemblies to reprogram cellular metabolism and signaling. Consequently, protein–peptide co-assembly represents a powerful strategy to modulate cell function beyond the limits of conventional molecular inhibition. By reorganizing the spatial arrangement of enzymes, receptors, and adaptor proteins, these assemblies reshape intracellular signaling landscapes, directing downstream responses such as apoptosis, proliferation, or differentiation. Coupling enzymatic catalysis, receptor engagement, or antibody recognition with peptide self-assembly allows precise control over the local distribution and interaction dynamics of biomolecules. Through this structural coupling, protein–peptide complexes can amplify, redirect, or attenuate signaling pathways in a programmable manner, endowing cells with emergent responsiveness to biochemical and mechanical stimuli. In essence, biomimetic peptide assemblies transform static molecular recognition into dynamic, systems-level regulation of cellular behavior (Table 2).

**Table 2:** Summary of intracellular peptide self-assembly interfacing with protein to modulate cellular function.

Name	Target sequence	Target protein	Location	Function	Ref.
Py^Me^Amp-BH3	GQVGRQLAIIGDDINR	Bcl-XL	Mitochondria outer membrane	Apoptosis	^76^
EIYsIYsE	GQVGRQLAIIGDDINR	Bcl-XL		Protein degradation	^77^
LP1	WTETGLTFTEKWNTDN	Hexokinase II		Apoptosis	^78^
FFY	FFY	Tubulin	Cytosol	Apoptosis	^80^
PyMT	Py-FFLLK	Tubulin		Apoptosis	^81^
NLS-FF-T	VKRKKKP	KPNA2		Metabolic inhibition	^84^
Gi-F-CAA	GACNWLPLYPCPV	GPX4		Ferroptosis	^85^
FGDF-Y^SO4^F	LTFGDFDEG	G3BP2		RNA metabolism	^86^
Pep-PEG	AWSATWSN_p_YWRH	CD47, CD24	Membrane	Immune activation	^88^
Py-FFK-BMS	Py-FFK-BMS	PD-L1		Protein degradation	^89^
PAD-1	KLARLLT	EGFR		Protein degradation	^90^
Anti-CD3-G7-RGD	Anti-CD3-GNNQQNYRGD	CD3 receptors, integrin α_v_β_3_		T cell activation	^91^
DMN-SIPL	LPVVLPI	Hepsin		Protein deactivation	^92^
NBD-ffs_p_y	ff	Fibronectin	Extracellular matrix	Cell adhesion	^95^

*Bcl-XL* B-cell lymphoma-extra large, *KPNA2* karyopherin subunit alpha-2, *GPX4* glutathione peroxidase 4, *G3BP2* Ras-GAP SH3 domain-binding proteins 2, *CD47* cluster of differentiation 47, *CD24* cluster of differentiation 24, *PD-L1* programmed death-ligand 1, *EGFR* epidermal growth factor receptor, *CD3* cluster of differentiation 3.

## Biomimetic peptide self-assembly interfacing with nucleic acid to regulate cellular signaling

**Fig. 6:**
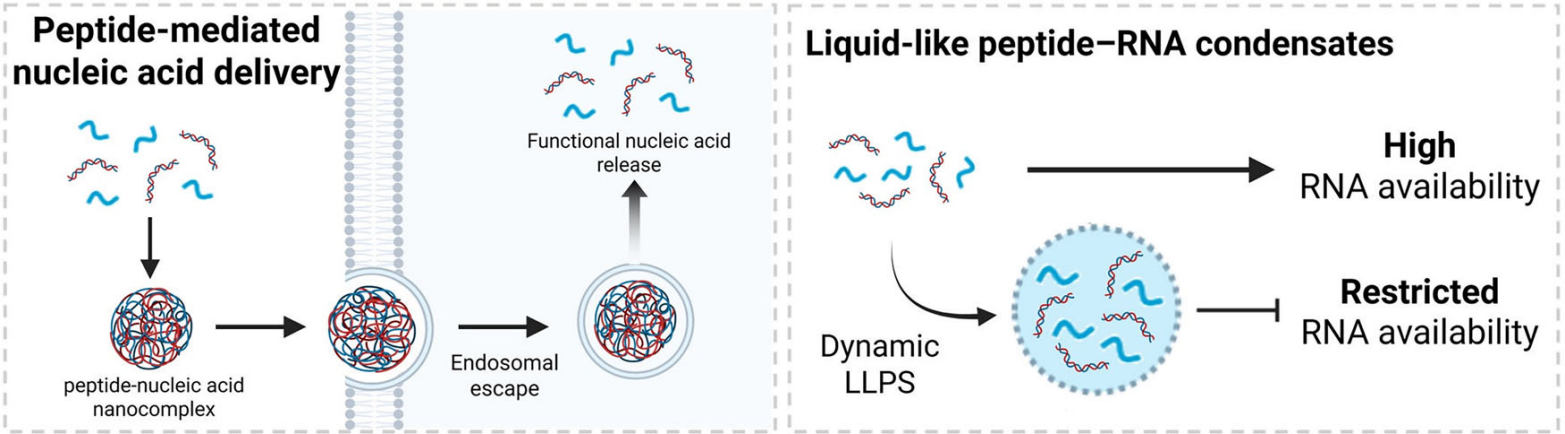
This schematic illustration represents biomimetic peptide self-assembly interfacing with nucleic acids to regulate cellular signaling. Peptide-nucleic acid nanocomplexes enable the intracellular delivery of functional nucleic acids through condensation, cellular uptake, and endosomal escape, ultimately facilitating cytosolic release and gene regulation. Moreover, peptide-RNA interactions can drive LLPS forming dynamic liquid-like condensates that modulate RNA sequestration and availability, thereby regulating RNA-associated cellular processes. LLPS liquid–liquid phase separation. The figure was created with BioRender.com.

Biomimetic peptide self-assemblies have emerged as dynamic molecular systems that mimic natural protein-folding and macromolecular organization to form functional supramolecular architectures. Through non-covalent interactions such as electrostatics, hydrogen bonding, hydrophobic packing, and π–π stacking, short peptides can organize into nanostructures capable of interacting with biomacromolecules^96^. Nucleic acids are particularly attractive binding partners because their negatively charged phosphate backbone and defined secondary structures permit multivalent complexation with cationic or aromatic peptide assemblies^97,98^. These hybrid systems reproduce the electrostatic complementarity seen in natural protein–nucleic acid complexes and provide a tunable platform for gene delivery, transcriptional modulation, and RNA-interference-based signaling^99^. Cooperative self-assembly between peptides and nucleic acids therefore offers a biomimetic bridge between molecular recognition and cellular communication^100^ (Fig. 6).

Importantly, the functional advantages of peptide–nucleic acid assemblies arise from multivalent and cooperative interactions enabled by spatial organization^9^. In contrast to monomeric peptides, which typically engage nucleic acids through weak and transient electrostatic contacts, supramolecular assemblies present multiple binding motifs in a confined nanoscale geometry, dramatically increasing local ligand density and effective binding avidity. Such multivalency allows simultaneous engagement of multiple phosphate groups along the nucleic acid backbone, resulting in enhanced complex stability even when individual interactions are relatively weak.

Moreover, cooperative binding within assembled structures reduces the entropic penalty associated with complex formation and promotes rebinding events following partial dissociation, thereby prolonging intracellular retention and functional activity. This collective stabilization effect distinguishes assembled peptides from their monomeric counterparts, which lack the spatial preorganization required for sustained nucleic acid association^17^. Consequently, peptide self-assembly transforms intrinsically modest molecular affinities into robust, tunable interactions capable of regulating nucleic-acid-mediated signaling processes.

The α-helical peptide KALA (WEAKLAKALAKALAKHLAKALAKALKACEA) demonstrates how sequence-encoded amphipathicity drives nucleic acid condensation and endosomal escape, thereby enhancing transfection efficiency^101^. A truncated analog, short-KALA3 (WEAKLAKALAKALA), preserves the helical motif and achieves efficient gene delivery as well as immune activation in dendritic cells^102^. Likewise, the amphipathic peptide CADY (Ac-GLWRALWRLLRSLWRLLWKA-Cya) self-assembles with small interfering RNA into “raspberry-like” nanoparticles that penetrate membranes directly, achieving potent sequence-specific knockdown^103^. Lipid-modified analogs such as PepFect-14 (Stearoyl-AGYLLGKLLOOLAAAALOOLL-NH₂; O = Orn) integrate hydrophobic acyl tails and cationic residues to generate stable small interfering RNA complexes that remain active even in human stem cells^104^. Meanwhile, classical cationic peptides including TAT (YGRKKRRQRRR) and R9 (RRRRRRRRR) demonstrate how simple arginine-rich motifs can self-assemble with nucleic acids, protect them from enzymatic degradation, and trigger RNAi-mediated signaling^105,106^. Beyond nanofibrous assemblies, charge-driven peptide–nucleic acid interactions can also yield liquid-like coacervate structures. For example, short cationic peptides such as RRASLRRASL undergo reversible complex coacervation with RNA through multivalent electrostatic interactions, forming membraneless condensates that dynamically sequester and concentrate nucleic acids in response to phosphorylation-dependent charge modulation^107^.

The versatility of peptide–nucleic acid assemblies is further exemplified by recent supramolecular systems that regulate intracellular nucleic acid dynamics. For example, nucleopeptide assemblies composed of thymine-capped D-nonapeptides, NP1 (thymine-ffkkfklkl), selectively sequester ATP in multidrug-resistant cancer cells, suppressing ATP-driven efflux pumps and enhancing the efficacy of doxorubicin chemotherapy^108^. These α-helical nanofibers undergo reversible assembly/disassembly in response to ATP/ADP conversion, mimicking actin-filament dynamics to regulate metabolic signaling. Similarly, assemblies of lysine-rich D-peptides, D-1 (NP1-NBD), interact with RNA to form membraneless condensates in the nucleolus, enabling selective organelle targeting and inducing DNA-damage-mediated apoptosis^109^. These findings show that nucleic acid-interfacing peptide assemblies can act as adaptive molecular condensates capable of modulating intracellular energy balance and gene expression through controlled non-covalent interactions.

Therefore, these examples collectively underscore how peptide self-assembly serves as a fundamental design principle for bridging supramolecular organization and genetic regulation. Through programmable non-covalent interactions, peptide–nucleic acid co-assemblies can dictate the condensation, intracellular trafficking, and selective release of nucleic acids with remarkable spatiotemporal precision. The emergent dynamic behaviors of these assemblies — ranging from ATP-responsive nucleopeptide fibers to RNA-condensing D-peptide condensates — highlight their ability to emulate natural biomolecular condensates and modulate gene-associated signaling in living systems. By rationally tuning sequence composition, charge distribution, and secondary-structure propensity, such biomimetic architectures hold great promise as modular platforms for therapeutic gene modulation, metabolic regulation, and regenerative medicine^110^ (Table 3).

**Table 3:** Summary of intracellular peptide self-assembly interfacing with nucleic acid to modulate cellular function.

Name	Target sequence	Target nucleic acid	Location	Function	Ref.
KALA	WEAKLAKALAKALAKHLAKALAKALKACEA	DNA/siRNA	Cytosol, endosomal membrane	Condensation, endosomal escape, enhanced gene transfection	^101^
Short-KALA3	WEAKLAKALAKALA	DNA/siRNA	Cytosol	Minimal helical motif maintaining immune activation	^102^
CADY	Ac-GLWRALWRLLRSLWRLLWKA-Cya	siRNA		Endocytosis-independent translocation, gene silencing	^103^
PepFect-14	Stearoyl-AGYLLGKLLOOLAAAALOOLL-NH_2_	siRNA		Stable nanocomplex formation, gene silencing in stem cells	^104^
TA	YGRKKRRQRRR	DNA/RNA	Cytosol, nucleus	siRNA delivery, gene silencing	^105^
R9	RRRRRRRRR	DNA/RNA	Cytosol	siRNA condensation, intracellular delivery	^106^
RNA/peptide coacervate	RRASLRRASL	RNA	Cytosol (condensate)	Sequestration, compartmentalization	^107^
NP1	Thymine-FFKKFKLKL	ATP	Cytosol	ATP sequestration, metabolic regulation, chemo-sensitization	^108^
D-1	Thymine-FFKKFKLKL-NBD	RNA	Nucleolus (condensate)	RNA interaction, nucleolar targeting, apoptosis	^109^

*DNA* deoxyribonucleic acid, *RNA* ribonucleic acid, *siRNA* small interfering RNA, *ATP* adenosine triphosphate.

## Conclusion and future perspectives

Peptide self-assembly has evolved from a fundamental supramolecular phenomenon into a practical strategy for modulating cellular signaling through biomimetic interfaces. As summarized in this Review, peptide assemblies can interact with lipid membranes, proteins, and nucleic acids to influence organelle function, PPIs, and gene expression. Their molecular programmability and structural adaptability enable precise control over spatial organization and dynamic responses within complex biological environments. These advances illustrate the growing potential of supramolecular chemistry to bridge molecular design and biological regulation.

Despite these promising developments, challenges remain in translating peptide assemblies into therapeutics or diagnostic tools. A deeper mechanistic understanding of intracellular assembly kinetics, interfacial energetics, and feedback with endogenous biomacromolecules is essential. In addition, developing quantitative imaging and computational methods to visualize and predict self-assembly processes in living systems will be crucial. Future research integrating sequence design, real-time imaging^8,95^, and artificial intelligence-based prediction^96–99^ may accelerate the creation of responsive and selective peptide assemblies tailored for disease-specific targets. Ultimately, biomimetic peptide self-assembly offers a versatile and expandable platform for developing next-generation therapeutic systems that can dynamically interface with living systems to restore or reprogram cellular function.
